# Anterior communicating artery complex fenestration combined with tandem aneurysm: a case report and literature review

**DOI:** 10.1097/MD.0000000000020013

**Published:** 2020-05-08

**Authors:** Haibing Liu, Jingfang Hong, Shousen Wang, Liangfeng Wei

**Affiliations:** a Department of Neurosurgery, Fuzong Clinical Medical College of Fujian Medical University, 900th Hospital; b Department of Neurosurgery, 900th Hospital, Dongfang Hospital, Xiamen University, Fuzhou, P.R. China.

**Keywords:** anterior communicating artery complex, coil embolization, fenestration deformity, subarachnoid hemorrhage, tandem aneurysm

## Abstract

**Introduction:**

Clinically, anterior communicating artery complex fenestration combined with fenestration-related aneurysms is rare, and combination of this condition with tandem aneurysms is even rarer.

**Patient concerns:**

A case of a 43-year-old man with spontaneous subarachnoid hemorrhage.

**Diagnosis:**

A computed tomography angiography examination revealed a fenestrated anterior communicating artery complex combined with 2 aneurysms. Then, a digital subtraction angiography examination was performed to further determine the diagnosis, which showed a complex anatomical structure of the local tissue. After the aneurysms ruptured, they were partially wrapped by a hematoma and compressed, which increased the difficulty of surgery.

**Interventions:**

An endovascular interventional therapy method was chosen, and a simple coil was successfully inserted through the blood vessel into the tandem aneurysms to maintain the integrity of the anatomical structure.

**Outcomes:**

The patient recovered well postoperatively. An imaging review after the operation did not show the aneurysms, and the upper and lower branches were patent.

**Conclusion:**

Therefore, endovascular treatment is an appropriate choice for arterial fenestration combined with tandem aneurysms, once the aneurysms have ruptured.

## 1 Introduction

The fenestration of an intracranial artery (FIA) is an anatomical variation with extremely low incidence. In this anatomical variation, a segment of a single vessel is divided into at least 2 channels, each consisting of an endothelial layer and a muscle layer (which may share the outer membrane), which merge into a single cavity along its longer distance.^[1,2]^ Sanders divided this condition into 3 types according to the location of fenestration^[3]^: near, middle and far fenestrated sections. Uchino divided the condition into 2 types according to the shape and size of the fenestration.^[4]^ In fissure-type fenestration, the blood vessels are short, the interval is not obvious, the local morphology is fissure-like or similar to a small hole, and the blood vessels resemble tumor-like expansion. In convex lens-type fenestration, the blood vessel is slightly larger, the interval is obvious, the partial shape is a fusiform or convex lens type, and the inner diameters of the 2 branch vessels of the fenestration opening might be uniform or inconsistent. The anterior communicating artery complex is the most frequent site of FIA.^[5]^ The association of FIA with aneurysms is rarely reported,^[6]^ and it is even rarer to have 2 aneurysms associated with the fenestration.^[7,8]^ This article reports a case of anterior communicating artery complex fenestration combined with 2 aneurysms in series, with a slender left A1 segment of the artery. Therefore, craniotomy or endovascular embolization would be very difficult.

## 2 Patient information

**Figure 1:**
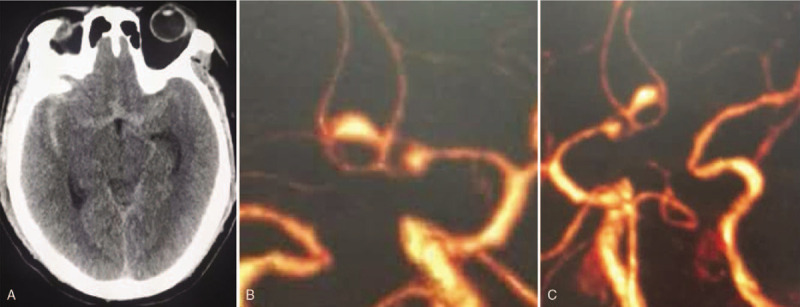
Preoperative head CTA examination. (A) The high-density shadow of the bilateral lateral fissure and the circle of Willis can be seen in the CT axial position, and the ventricle is not significantly enlarged. (B), (C) The anterior communicating artery complex fenestration before revascularization; 2 tandem aneurysms were located at the fenestration, and the right A1 artery is not visible. CTA = computed tomography angiography.

**Figure 2:**
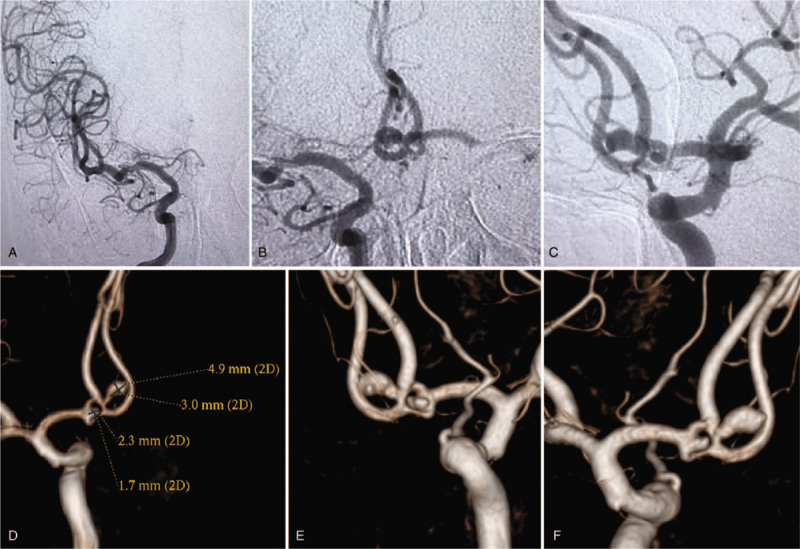
Preoperative head DSA examination. (A) An angiographic examination of the right internal carotid artery showed that the right side A1 segment was slender. (B) The left common carotid artery was obstructed, and right internal carotid artery angiography showed that the right A1 segment was normal. The bilateral A2 blood flow could still be compensated, and the blood flow direction was toward the left A1 countercurrent. (C) Angiography of the left internal carotid artery showed anterior communicating artery complex fenestration, and aneurysms were present at the proximal and distal ends of the fenestration. (D) After reconstruction by 3-dimensional DSA, the size of the 2 aneurysms was measured. (E, F) After 3-dimensional reconstruction, a small break in the distal end of the distal aneurysm could be seen, and there were 2 lesions at the proximal aneurysm. DSA = digital subtraction angiography.

Patient Chen ××, male, 43-years-old, had sudden onset of loss of consciousness with severe headache 6 hours after vomiting. The patient was referred to our department and had undergone a computed tomography scan at the local hospital, which suggested subarachnoid hemorrhage (SAH). A physical examination showed the following: lethargy, slight agitation, and the patient can make right answers to relevant questions. The bilateral pupils of the patient were equally large, round and approximately 3.0 mm in diameter. Direct and indirect sensitivity to light reflection was observed, neck resistance was obvious, and the muscle strength and muscle tension of the limbs were normal. An auxiliary examination showed the following: a computed tomography angiography (CTA) scan of the head showed high-density areas in the circle of Willis, bilateral lateral fissure pool, and anterior longitudinal fissure. A SAH was considered, and the right A1 segment of the artery was not visible. The anterior communicating artery complex fenestration incorporated 2 saccular tumor-like projections (Fig. 1). A digital subtraction angiography (DSA) examination under general anesthesia with endotracheal intubation of the patient was conducted. The right A1 segment of the artery was present, and the anterior communicating artery complex fenestration exhibited aneurysms at both the proximal and distal ends. The proximal aneurysm was approximately 2.3 mm × 1.7 mm in size, and the distal aneurysm was approximately 4.9 mm × 3.0 mm (Fig. 2). We placed the head of a 6F guide catheter (Envoy, Codman) in the ascending section of the left internal carotid artery. Using a Synchro-14 (Stryker Neurovascular) micro-guidewire, we placed a straight Echelon-10 catheter (Micro Therapeutics Inc. dba ev3 Neurovascula) into the distal aneurysm through the main site of the fenestration.

**Figure 3:**
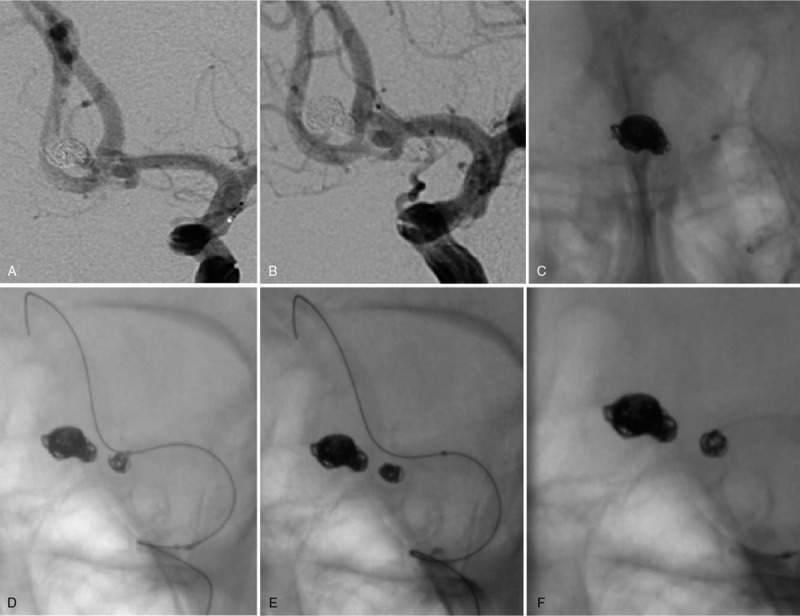
During surgery. (A) The microcatheter was delivered into the aneurysm using a guide wire through the left internal carotid artery, and the spring coil was placed. (B, C) After using a simple coil to embolize the aneurysm at the bottom, the aneurysm was not visible on angiography images, and the microcatheter was withdrawn. (D) Because the aneurysm in the near-angle area was a wide-neck aneurysm, after placing the coil, to prevent the coil from slipping off, the microcatheter was withdrawn, and the micro-guide wire was moved to the distal end of the anterior cerebral artery with using the guide wire. (E) The spring coil did not contact the guide wire. (F) After the position of the spring coil was stable, the guide wire was withdrawn, and the shape of the spring coil was observed to be very stable.

After filling with an Axium 5 × 15 cm coil, Axium 3 × 8 cm, 3 × 8 cm, 2 × 6 cm, and 2 × 4 cm coils were used for tamping, and angiography did not show the distal aneurysms. The images of the upper and lower main branches of the fenestration were clear. Then, we pulled the straight Echelon 10 microcatheter back and entered the proximal aortic aneurysm using a Synchro micro-guidewire. After using an Axium 2 × 3 cm coil, the near and distal aneurysms were absent on angiographic examination. The upper and lower main arteries showed clear images. The spring coil was released to prevent the spring ring from slipping due to the wide neck of the aneurysm. Then, the Echelon 10 microcatheter was replaced with a Synchro micro-guidewire, and the Echelon 10 microcatheter was withdrawn from the aneurysm. The Synchro microwire was moved to the A3 segment on the same side, and the Echelon 10 microcatheter was completely withdrawn from the aneurysm neck. We monitored the procedure to that the coil did not contact the Synchro micro-guidewire. After confirming that the coil was stable in the tumor cavity, the Echelon 10 microcatheter and the Synchro micro-guide wire were completely removed (Fig. 3). After the operation, the patient underwent continuous lumbar puncture to release the bloody cerebral effusion. After 7 days, the headache and dizziness symptoms were relieved, and the patients were discharged. The patient did not have loss of speech or the visual field or physical dysfunction. Head magnetic resonance angiography (MRA) and DSA results were reviewed at 3 and 6 months after surgery. The images of the anterior and posterior regions of the communicating artery complex with fenestration were good, and both aneurysms were absent in the images.

**Figure 4:**
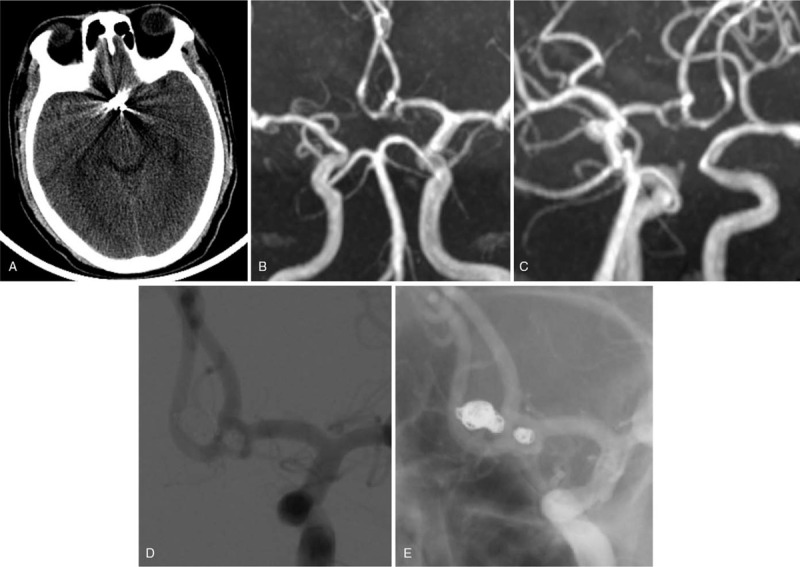
Review after surgery. (A) Postoperative head CT review showed a metal artifact in front of the brain stem, and no ischemic lesions or expansion of the ventricles was observed. (B, C) At 3 mo after the operation, an MRA examination showed that the anterior and posterior main blood flow of the anterior communicating artery complex fenestration was smooth, and there was no obvious tumor-like bulge. (D, E) A DSA examination of the head at 6 mo after surgery indicated that the anterior communicating artery complex fenestration had a smooth upper and lower trunk, and the 2 aneurysms were not displayed in the images. CT = computed tomography, DSA = digital subtraction angiography, MRA = magnetic resonance angiography.

No obvious ischemic change in the brain tissue associated with the blood supply to the anterior cerebral artery was noted in the images (Fig. 4).

## 3 Discussion

### 3.1 The incidence and characteristics of intracranial artery fenestration with aneurysm

The most common anatomical variations of intracranial arteries include variations in the circle of Willis, cervical-vertebral basilar artery anastomosis, fenestration, repetition, and other vascular anatomical anomalies found at the skull base. Fenestration was previously described as the division of the arterial lumen so that a single blood vessel creates multiple channels. However, the vessel should more accurately be referred to as “partially non-fused,” which refers to a single originating vessel with at least 2 channels anywhere along its path, with each channel consisting of an endothelial layer and a muscle layer (which might share the outer membrane). During the course of the disease, the vessels merge into a single cavity.^[9–11]^ Intracranial artery fenestration is caused by a failure of a pair of primitive embryonic vascular fusions or an incomplete anastomosis of the original vascular network.^[12]^ It involves the local replication of vascular endothelial cells, the division of the arterial lumen, including the division of endothelial cells and the media, and in some cases, the division of the outer membrane.^[13]^

The actual incidence of FIA is difficult to determine. The incidence varies greatly depending on the method of examination. According to surgical anatomy, DSA, CTA, and MRA examinations, the difference in the incidence upon window examination varies from 0.7% to 60%.^[3]^ FIA is common in the area of the anterior communicating artery complex, followed by the vertebral basilar artery system and the middle cerebral artery region.^[14]^ According to microsurgery results, the incidence of anterior communicating artery complex fenestration is approximately 40%, while angiographic examinations have found that the incidence of fenestration is lower. The most common region of fenestration in autopsy studies is the anterior communicating artery area, and only 5% of cases are confirmed by angiography.^[6]^ Cooke et al^[1]^ examined 10,927 patients by DSA and found 228 cases (2.1%) of intracranial fenestration. Posterior circulation (73.2%) fenestration was more common than anterior circulation fenestration (24.6%). There was no significant correlation between the incidence of fenestration and gender.

Controversy exists regarding whether FIA is related to aneurysm formation. One hypothesis states that eddy currents cause defects in the medial lining of the proximal and distal vascular walls of the fenestration, leading to the occurrence of aneurysms. Related research has shown that this type of FIA includes obvious intravascular eddy currents, which markedly thin the intima of the blood vessels, thus significantly increasing the chance of formation of cerebral aneurysms.^[15]^ In addition, studies^[16]^ have shown that FIA is accompanied by a higher incidence of aneurysm because the defects in the middle layer of the vessel wall are accompanied by an increase in hemodynamic pressure.

Sun et al^[17]^ analyzed the MRA results of 4652 patients with cerebrovascular disease and found that intracranial artery fenestration was associated with aneurysms; 141 patients had FIA and 24 patients were diagnosed with intracranial aneurysm. The incidence rate was 17.0%, which was significantly higher than that of non-FIA complicated aneurysms. Van Rooij et al^[2]^ found no significant difference in the incidence of arterial fenestration between patients with and without aneurysms. Although nearly one-third of fenestrations were anatomically associated with aneurysms, these aneurysms were in the anterior communicating artery complex. This observation might have occurred due to the use of a specific patient population with suspected aneurysms because most fenestrations and aneurysms occur in the anterior communicating artery complex. The exact link between aneurysm and fenestration at this location is unclear. Sogawa et al^[18]^ analyzed the MRA results of 16, 416 patients and found that 212 patients had 215 fenestrations; 13 aneurysms were found in 9 patients. No aneurysm was located at the fenestration. Therefore, they concluded that there was no significant difference in the incidence of aneurysms between patients with fenestrations and the general population. Similarly, Bharatha et al^[14]^ used CTA to examine 504 patients and found no significant difference in the incidence of fenestration between patients with and without aneurysms. De Gast et al^[6]^ used 3-dimensional angiography to analyze the correlation between anterior communicating artery complex fenestration and aneurysm and found no definite correlation between these factors.

### 3.2 Characteristics of this case

Based on the relationship between FIA and aneurysm, van Rooij et al^[2]^ divided aneurysms into 3 types. Type I is an aneurysm located at the proximal end of the fenestration. Type II is an aneurysm located at the window opening. Type III is a case with no correlation between the aneurysm and the fenestration position. In the present case, the anterior communicating artery complex exhibited a fenestration-like triangle, and aneurysms were present at the apex angle and bottom of the artery. The tumor grew in the direction of blood flow, the blood vessels on both sides were slender, the underlying aneurysm and the fenestration branch were “Z-shaped,” and there was an obvious ladder (Fig. 2D). The insertion of microcatheter into the aneurysm cavity was difficult. After the microcatheter was bent into a “Z-shape,” it was positioned in the aneurysm cavity, and the aneurysm was then embolized with a simple coil. The aneurysm in the apex area was irregular and had a lobulated shape. It could not be determined whether the 2 aneurysms (Fig. 2E) were unruptured; therefore, they were not treated at that time. However, the aneurysm had a wide neck, and after releasing the coil, the Synchro micro-guide wire was fed through the Echelon 10 microcatheter. Then, the Echelon 10 microcatheter was withdrawn from the aneurysm, and the synchro micro-guide wire was moved to the ipsilateral A3 segment. Next, the Echelon 10 microcatheter was completely removed from the neck, and the coil was observed to have no contact with the Synchro micro-guidewire. After the coil was stabilized in the aneurysm cavity, the Echelon 10 microcatheter and the synchro micro-guidewire were completely withdrawn. If the coil cannot be fixed, bracket assistance would be necessary. Because the upper and lower trunks were thin at the opening of the artery, it was highly probable that use of a stent could block the trunk. In the case of aneurysm rupture, vasospasm was likely to cause bilateral anterior cerebral artery ischemia or occlusion.

### 3.3 Treatment characteristics of aneurysms fenestration

Intracranial aneurysm rupture often causes SAH, and after conservative medical treatment, the mortality rate is approximately 60% within 6 months. Neurosurgical clipping or endovascular embolization are necessary, and for both surgical clipping and spring coil embolization (no stent or balloon), endovascular embolization is preferred.^[19,20]^ In the absence of a significant occupying effect of an aneurysm or hematoma, endovascular interventional embolization and craniotomy are suitable for aneurysms of the anterior communicating artery complex. The 2 aneurysms were located in proximal area and the bottom area of the fenestration of the anterior communicating artery complex. Due to the complex geometry of the fenestration and aneurysms, it would be very difficult to obtain sufficient visualization of the aneurysm neck during surgery, and it would be easy to damage the important perforating vessels of the anterior communicating artery complex.^[21]^

### 3.4 Treatment selection

It is appropriate to choose endovascular interventional embolization to treat an anterior communicating artery complex with associated aneurysms. Because the vascular structure of the window opening is highly complicated, performing a craniotomy to obtain clear anatomical exposure would inevitably increase the damage, and it would be easy to damage the normal vessels during clipping. However, using 3-dimensional reconstruction, endovascular interventional embolization can obtain good blood vessel images, and the possibility of occlusion is decreased. With the advancement of intravascular interventional embolization technology and the continuous development of materials, the success rate and safety of interventional embolization for intracranial aneurysms had been greatly improved. Endovascular embolization of intracranial aneurysms could effectively prevent rupture of intracranial aneurysms, and the trauma to the patient is minor, resulting in an easy recovery.

A single fenestration of the anterior communicating artery complex combined with related aneurysms at the anterior and posterior end of the fenestration is extremely rare; endovascular interventional embolization is the first safe and effective treatment option for this condition. During vascular interventional embolization, 3-dimensional rotational angiography could be used to completely observe the relationship between the aneurysms and surrounding blood vessels, select the best treatment angle for embolization, and maximize the protection of related blood vessels.

## Acknowledgment

We would like to express our gratitude to all those who helped us during the writing of this paper.

## Author contributions

**Conceptualization:** Haibing Liu, Jingfang Hong.

**Data curation:** Haibing Liu, Liangfeng Wei.

**Formal analysis:** Haibing Liu, Shousen Wang.

**Writing – original draft:** Haibing Liu.

**Writing – review & editing:** Haibing Liu, Jingfang Hong.
